# Activation of toll like receptor 4 attenuates GABA synthesis and postsynaptic GABA receptor activities in the spinal dorsal horn via releasing interleukin-1 beta

**DOI:** 10.1186/s12974-014-0222-3

**Published:** 2015-01-09

**Authors:** Xisheng Yan, Enshe Jiang, Han-Rong Weng

**Affiliations:** Department of Pharmaceutical and Biomedical Sciences, The University of Georgia College of Pharmacy, 240 West Green Street, Athens, GA 30602 USA; Department of Pain Medicine, University of Texas MD Anderson Cancer Center, Houston, TX 77030 USA; Department of Cardiovascular Medicine, the Third Hospital of Wuhan, Wuhan, Hubei Province China; Institute of Public Hygiene, Henan University Nursing School, Kaifeng, China

**Keywords:** Nociception, GLT-1, Neuroinflammation, Cytokines

## Abstract

Toll like receptor 4 (TLR4) is an innate immune pattern recognition receptor, expressed predominantly on microglia in the CNS. Activation of spinal TLR4 plays a critical role in the genesis of pathological pain induced by nerve injury, bone cancer, and tissue inflammation. Currently, it remains unknown how synaptic activities in the spinal dorsal horn are regulated by TLR4 receptors. Through recording GABAergic currents in neurons and glial glutamate transporter currents in astrocytes in rodent spinal slices, we determined whether and how TLR4 modulates GABAergic synaptic activities in the superficial spinal dorsal horn. We found that activation of TLR4 by lipopolysaccharide (LPS) reduces GABAergic synaptic activities through both presynaptic and postsynaptic mechanisms. Specifically, LPS causes the release of IL-1β from microglia. IL-1β in turn suppresses GABA receptor activities at the postsynaptic site through activating protein kinase C (PKC) in neurons. GABA synthesis at the presynaptic site is reduced upon activation of TLR4. Glial glutamate transporter activities are suppressed by IL-1β and PKC activation induced by LPS. The suppression of glial glutamate transporter activities leads to a deficiency of glutamine supply, which results in an attenuation of the glutamate-glutamine cycle-dependent GABA synthesis. These findings shed light on understanding synaptic plasticity induced by activation of TLR4 under neuroinflammation and identify GABA receptors, glial glutamate transporters, IL-1β and PKC as therapeutic targets to abrogate abnormal neuronal activities following activation of TLR4 in pathological pain conditions.

## Introduction

Toll like reeptor 4 (TLR4) is an innate immune pattern recognition receptor, expressed predominantly on microglia in the CNS [1,2]. Activation of spinal TLR4 is critical to the genesis of many pathological pain conditions like those induced by nerve injury [3-5], bone cancer [6], peripheral tissue inflammation induced by complete Freund’s adjuvant [7], and opioid-induced hyperalgesia [8]. Despite the extensive studies of the role of spinal TLR4 in spinal nociceptive processing, little is known about how spinal synaptic activities are regulated by TLR4.

It is generally believed that pathological pain is a reflection of aberrant neuronal activities along the pain signaling pathway, which includes primary sensory neurons in the periphery, neurons in the spinal dorsal horn, and supraspinal areas in the central nervous system (CNS) [9]. Multiple factors can contribute to aberrant neuronal activities in the spinal dorsal horn, including changes in the balance between presynaptic excitatory and inhibitory input, the active and passive electrophysiological membrane properties of the dorsal horn neurons, and functions of postsynaptic excitatory and inhibitory ligand gated ion channels [10,11]. GABAergic synapses are the major inhibitory synapses in the CNS. Gamma-amino butyric acid (GABA) released from GABAergic interneurons inhibits neuronal activities through acting on GABA receptors at presynaptic terminals to reduce release of excitatory neurotransmitter (like glutamate), or acting on postsynaptic GABA receptors in neurons to produce inhibitory postsynaptic currents (IPSCs), or membrane hyperpolarization, in postsynaptic neurons [12]. Reduced neuronal inhibition, termed 'disinhibition', resulting from decreased GABAergic synaptic strength in the spinal dorsal horn is a crucial mechanism contributing to the development and maintenance of pathological pain [13-15]. Weakening of GABAergic synaptic strength may result from a reduction of the GABA transmitter release probability at the presynaptic site, attenuation of the GABA receptor function at the postsynaptic neurons, or impairment in GABA synthesis at the presynaptic site.

GABA synthesis through the glutamate-glutamine cycle is an important mechanism maintaining the GABA homeostasis [16-19]. The glutamate-glutamine cycle takes place between neurons and astrocytes. Extracellular glutamate is taken up by glial glutamate transporters into an astrocyte. Inside the astrocyte, glutamate is transformed to glutamine via glutamine synthetase. The astrocyte then exports glutamine outside the cell, where glutamine is taken up by neurons. Phosphate-activated glutaminase in neurons deaminates glutamine, producing glutamate. Glutamate in GABAergic neurons is then decarboxylated by glutamic acid decarboxylase (GAD) to become GABA. Finally, GABA is taken up into synaptic vesicles by vesicular GABA transporters, completing the cycle. It is unknown whether and how GABA synthesis is regulated by the TLR4 signaling in the spinal dorsal horn.

In this study, using whole cell voltage-clamp recording techniques we, for the first time, demonstrated that GABAergic synaptic activities in the superficial spinal dorsal horn are suppressed upon the activation of TLR4 by lipopolysaccharide (LPS). We also revealed the signaling molecules (IL-1β, protein kinase C (PKC), glutamate transporters) mediating the effects induced by LPS on GABAergic synaptic strength at the presynaptic and postsynaptic sites. Our study provides therapeutic targets for the normalization of altered synaptic activities following activation of TLR4.

## Materials and methods

All experiments were approved by the Institutional Animal Care and Use Committees at the University of Georgia and the University of Texas MD Anderson Cancer Center, and were fully compliant with the National Institutes of Health Guidelines for the Use and Care of Laboratory Animals.

### Spinal slice preparation

Young adult (6 to 8 weeks old) male Sprague–Dawley rats or glial fibrillary acidic protein-GFP (GFAP-GFP) transgenic mice with FVB/N genetic background were used. GFAP-GFP transgenic mice were obtained from the Jackson Laboratory (Bar Harbor, ME, USA). The rodents were deeply anesthetized using inhaled isoflurane, and a laminectomy was then performed to remove the lumbar spinal cord. The lumbar spinal cord section was placed in ice-cold sucrose-based artificial cerebrospinal fluid (aCSF) pre-saturated with 95% O_2_ and 5% CO_2_. The sucrose-based aCSF contained 234 mM sucrose, 3.6 mM KCl, 1.2 mM MgCl_2_, 2.5 mM CaCl_2_, 1.2 mM NaH_2_PO_4_, 12.0 mM glucose, and 25.0 mM NaHCO_3_. The pia-arachnoid membrane was removed from each section. The L4-5 spinal segment, identified by the lumbar enlargement and large dorsal roots, was attached with cyanoacrylate glue to a cutting support, which was then glued onto the stage of a vibratome (Series 1000, Technical Products International, St. Louis, MO, USA). Transverse spinal cord slices (400 μm thick) were cut in the ice-cold sucrose aCSF and then pre-incubated for at least 2 hours in Krebs solution oxygenated with 95% O_2_ and 5% CO_2_ at 35°C before they were transferred to the recording chamber. The Krebs solution contained 117.0 mM NaCl, 3.6 mM KCl, 1.2 mM MgCl_2_, 2.5 mM CaCl_2_, 1.2 mM NaH_2_PO_4_, 11.0 mM glucose, and 25.0 mM NaHCO_3_.

### Recordings of GABAergic currents from rat spinal slices

Following pre-incubation, a spinal slice was placed in the recording chamber (1.5 ml in volume), perfused with Krebs solution at 35°C, and saturated with 95% O_2_ and 5% CO_2_. Borosilicate glass recording electrodes (resistance, 3 to 5 MΩ) were made and filled with an internal solution containing 110 mM Cs_2_SO_4_, 5 mM KCl, 2.0 mM MgCl_2_, 0.5 mM CaCl_2_, 5.0 mM HEPES, 5.0 mM ethylene glycol tetraacetic acid (EGTA), 5.0 mM ATP-Mg, 0.5 mM Na-GTP, and 10 mM lidocaine N-ethyl bromide (QX314), adjusted to pH 7.2 to 7.4 with 1 M CsOH (290 to 300 mOsm) [20]. The recording electrodes were directed to the spinal dorsal horn lamina II area. Whole-cell configurations were established by applying moderate negative pressure after electrode contact [21]. A seal resistance of ≥ 2 GΩ and an access resistance of about 20 MΩ were considered acceptable. GABAergic currents were recorded in the presence of 0.5 μM strychnine (a glycine receptor inhibitor), 10 μM DNQX (an AMPA/kainate receptor inhibitor) and 25 μM D-AP5 (N-methyl-D-aspartate (NMDA) receptor inhibitor) at a holding potential of 0 mV [15]. When miniature GABAergic IPSCs (mIPSCs) were recorded, tetrodotoxin (TTX, 1 μM) was further added into the recording bath. The currents recorded under such condition were completely abolished by the classic antagonist of GABA_A_ receptors, bicuculline (10 μM) [20]. To evoke GABAergic IPSCs, neurons in the spinal lamina II area were stimulated by a rectangular electrical pulse (0.1 ms, 0.5 mA, repeated every 40 seconds) delivered through a concentric bipolar stainless electrode (50 μm in diameter, isolated except for the tip) placed within 150 μm of the recorded neurons in the dorsal horn [20,22]. Only monosynaptic GABAergic IPSCs were recorded. Identification of IPSCs as monosynaptic was based on a constant latency with graded intensity and high-frequency repetitive stimulation (20 Hz) [20,23]. In some experiments, GABAergic currents were evoked by puffing 100 μM GABA onto the recorded neuron through a glass pipette connected to a Picospritzer (Parker Hannifin Precision Fluidics Division, Hollis, NH, USA) controlled by a computer. The puffing pipette tip (about 15 μm), the pipette opening tip (3 to 4 μm), the puffing pressure (3 psi), the puffing duration (25 ms, repeated every 30 seconds) [24], and the depth of the cell in the slice (about 50 μm below the surface) were kept constant across all experiments.

### Recordings of glial glutamate transporter currents (GTCs) from astrocytes in the spinal dorsal horn

GTCs were recorded from GFAP-GFP transgenic mice as we described previously [25]. In these mice, GFP under the control of the astrocyte-specific GFAP promoter was overexpressed. Astrocytes in these mice were easily identified by the expression of GFP under a fluorescent microscope. To record GTCs, a mouse spinal slice was placed in a recording chamber perfused with Krebs solution. Visualized whole-cell patch clamp recordings were obtained from the spinal dorsal horn lamina II astrocytes identified by GFP under the fluorescent microscope. Borosilicate glass recording electrodes (resistance, 4 to 6 MΩ) were filled with 145 mM potassium gluconate, 5 mM NaCl, 1 mM MgCl_2_, 0.2 mM EGTA, 10 mM HEPES, 2 mM Mg-ATP and 0.1 mM Na-GTP (pH 7.3, 290 to 300 mOsm). GTCs were recorded at a holding potential of −80 mV in voltage clamp mode in the presence of blockers of GABA_A_ receptors (10 μM bicuculline), glycine receptors (5 μM strychnine), AMPA/kainate receptors (10 μM DNQX), NMDA receptors (25 μM D-AP5), and tetrodotoxin (1 μM) at 35°C. GTCs were evoked by puffing 50 μM L-glutamate onto the recorded astrocyte through a glass pipette connected to a Picospritzer controlled by a computer using the same parameters as those used for evoking GABAergic currents. Access resistance within the range of 20 MΩ was monitored continuously throughout the experiments. Recordings were abandoned when the access resistance changed more than 20% [25].

### Materials

Tetrahydropyridin-4-yl methylphosphinic acid (TPMPA), strychnine, DNQX and D-AP5, GF 109203X were purchased from Tocris Bioscience (Park Ellisville, MO, USA). LPS, minocycline, TTX, glutamine, GABA and phorbol 12-myristate 13-acetate (PMA) were obtained from Sigma (St. Louis, MO, USA). Recombinant human IL-1β and IL-1ra proteins were purchased from R&D Systems (Minneapolis, MN, USA). PKCI 19–31 was obtained from EMD Biosciences (San Diego, CA, USA). All pharmacological agents were applied by perfusion into the recording chamber unless indicated.

### Data analysis

Data were recorded with Axopatch 700B amplifiers (Molecular Devices, Sunnyvale CA, USA), digitized at 10 kHz and analyzed offline. Four GABAergic evoked IPSCs (eIPSCs) or GTCs at baseline, in the presence, and after washout of tested drugs were averaged. Clampfit 10.2 software (Molecular Devices, Sunnyvale CA, USA) was used to measure the amplitude of eIPSCs and GTCs. The frequencies and amplitudes of mIPSCs were analyzed with MiniAnalysis software (synaptosoft, NJ, USA). Data are presented as means ± standard errors (SE). One-way analysis of variance (ANOVA) with repeated measures followed by Bonferroni’s *post hoc* test was used to determine statistical differences in data collected in three groups while the paired Student’s *t-*test was used to determine statistical differences for data collected in twogroups. A *P-*value < 0.05 was considered statistically significant.

## Results

### LPS suppresses GABAergic IPSCs through activating microglia

**Figure 1:**
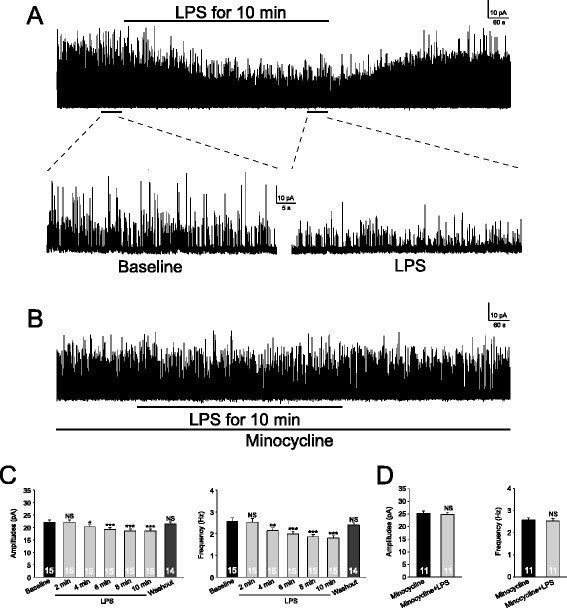
**Lipopolysaccharide (LPS) suppresses GABAergic inhibitory postsynaptic currents (IPSCs) through activating microglia.** GABAergic miniature IPSCs (mIPSCs) recordings obtained from superficial spinal dorsal horn neurons before, during and after washout of LPS (1 μg/ml) in the absence **(A)** and presence **(B)** of minocycline (100 μM) are shown. **(C)** shows the mean (+SE) GABAergic mIPSC amplitude and frequency before (baseline), during and after washout of LPS. The mean amplitude and frequency in 2-minute bins during LPS perfusion are displayed. **(D)** shows the mean (+SE) GABAergic mIPSC amplitude and frequency before and during perfusion of LPS in the presence of minocycline. Number of neurons included in each group for the analysis is shown in each bar. *P < 0.05; **P < 0.01; ***P < 0.001; NS: no statistical significance.

To study the impact of TLR4 activation on GABAergic synaptic activities, GABAergic mIPSCs were first recorded from neurons located in spinal lamina II in rats in the presence of 1 μM TTX before and after bath perfusion of the well-known TLR4 agonist LPS (1 μg/ml) [26]. As shown in Figure 1A, LPS significantly reduced mIPSC amplitudes and frequencies at the same time. These effects appeared at 4 minutes from the beginning of LPS perfusion and plateaued between 6 to 8 minutes. At 10 minutes after the perfusion, LPS significantly reduced the mean mIPSC amplitudes by 15.35 ± 1.45% (n = 15, *P* < 0.001) and mean mIPSC frequencies by 29.53 ± 2.91% (n = 15, *P* < 0.001). Similarly, when neurons in the same area in mice were recorded, LPS (1 μg/ml) significantly reduced the mean mIPSC amplitudes by 14.69 ± 3.28% (n = 7, *P* < 0.01) and frequencies by 27.11 ± 2.36% (n = 7, *P* < 0.001). It is generally believed that TLR4 is predominantly expressed in microglia [1,2,27]. If this is the case, the effects induced by LPS on mIPSCs should be abolished when activation of microglia is blocked. Previous studies have shown that minocycline inhibits the production of immune activators by macrophages, microglia [28,29], and monocytes [30]. More specifically, minocycline has been shown to specifically suppress microglial activation in the spinal dorsal horn at the concentration of 100 μM [31]. We then perfused minocycline (100 μM) into the recording for 10 minutes prior to addition of LPS into the bath. In the presence of minocycline, LPS no longer altered the amplitudes or frequencies of GABAergic mIPSCs recorded from rats (Figure 1B) and mice (amplitudes: n = 7, *P =* 0.82; frequencies: n = 7, *P =* 0.55, data not shown). Previous studies show that intraperitoneal injection of minocycline (40 mg/kg) suppresses phosphorylation of extracellular signal regulating kinases (ERK) in spinal dorsal horn neurons induced by intraperitoneal injection of acetic acid [32]. To confirm that the minocycline treatment under our experimental conditions did not alter the GABAergic synaptic activities, we examined the effects of minocycline on GABAergic mIPSCs. We found that perfusion of minocycline at 100 μM for 20 minutes did not significantly alter the amplitudes (*P =* 0.62) or frequencies (*P =* 0.52) of mIPSCs recorded from neurons in mice (n = 5). These data indicate that LPS suppresses GABAergic synaptic activities through activation of microglia. Several mechanisms may underlie the LPS-induced attenuation of GABAergic synaptic activities, which at least include (a) a reduced release probability of GABA at presynaptic terminals; (b) a decreased function of GABA receptors at postsynaptic neurons; and (c) the decreased synthesis of GABA. We conducted the following experiments to determine which of these mechanisms contributes to the changes of GABAergic synaptic activities induced by LPS.

### LPS reduced neuronal GABA receptor activities through releasing IL-1β and activation of PKC

**Figure 2:**
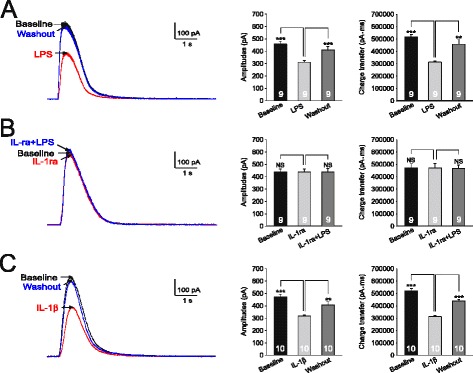
**Lipopolysacharide (LPS) suppresses GABAergic currents via releasing IL-1β. (A)** shows recordings of GABAergic currents evoked by gamma-amino butyric acid (GABA) (100 μM) injected onto the recorded neuron by a puff-electrode at baseline, during and after washout of LPS (1 μg/ml). **(B)** shows recordings of GABA currents evoked by GABA (100 μM) injected onto the recorded neuron at baseline, during bath-perfusion of the IL-1β receptor blocker (IL-1ra, 100 ng/ml), and further addition of LPS (1 μg/ml). **(C)** shows recordings of GABA currents evoked by GABA (100 μM) injected onto the recorded neuron at baseline, during and after washout of IL-1β (10 ng/ml). The mean (+SE) amplitudes of GABAergic currents and charge transfers at baseline, during and after washout of each tested agent are shown in bar graphs. Number of neurons included for the analysis is shown in each bar. **P < 0.01; ***P < 0.001; NS: no statistical significance.

To determine whether the function of postsynaptic GABA receptors is altered during perfusion of LPS, we recorded GABAergic currents evoked by exogenous GABA injected onto the recorded neuron through a puffing glass pipette (GABA concentration: 100 μM; duration: 25 ms). As shown in Figure 2A*,* GABAergic currents evoked by exogenous GABA (n = 9, *P* < 0.001) were significantly reduced during perfusion of LPS, indicating that activation of TLR4 results in attenuation of neuronal GABA receptor activities under our experimental conditions. It has been known that IL-1β is released from microglia activated by LPS [27] and IL-1β receptors are expressed in spinal dorsal horn neurons [33]. We tested if IL-1β mediates the effects induced by LPS on neuronal GABA currents. After recording baseline GABA currents evoked by GABA injected onto the recorded neuron through the puffing glass pipette, we perfused the IL-1β antagonist (IL-1ra, 100 ng/ml) into the recording chamber and re-recorded GABA. GABA currents were not altered by IL-1ra perfusion (Figure 2B), indicating that under normal conditions, activities of GABA receptors in neurons are not under the control of endogenous IL-1β. In the presence of IL-1ra (100 ng/ml) further addition of LPS (1 μg/ml) into the bath did not alter GABA current areas and amplitudes (Figure 2B), indicating that IL-1β mediates the inhibitory effects induced by LPS on neuronal GABA receptor activities. Further, perfusion of IL-1β (10 ng/ml) into the recording bath significantly reduced GABAergic currents (Figure 2C), consistent with a previous report [34]. Together, these data indicate that LPS suppresses neuronal GABA receptor activities via releasing IL-1β.

**Figure 3:**
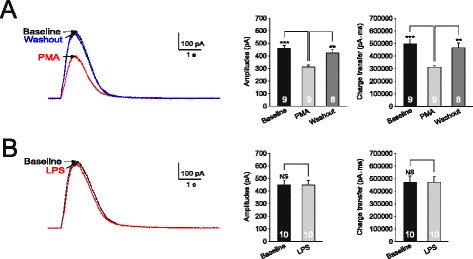
**Lipopolysaccharide (LPS) suppresses GABAergic currents via activating protein kinase C (PKC). (A)** shows recordings of GABAergic currents evoked by gamma-amino butyric acid (GABA) (100 μM) injected onto the recorded neuron at baseline, during and washout of the PKC activator (PMA, 2 μM). **(B)** shows recordings of GABA currents evoked by GABA (100 μM) injected onto a neuron recorded with a recording pipette filled with the intracellular solution containing the PKC inhibitor (PKCI 19–30, 5 μM) at before (baseline), and during perfusion of LPS (1 μg/ml). The mean (+SE) amplitudes of GABAergic currents and charge transfers at baseline, during and after washout of each tested agent are shown in bar graphs. Number of neurons included for the analysis is shown in each bar. **P < 0.01; ***P < 0.001; NS: no statistical significance.

We recently showed that PKC is an important kinase activated by IL-1β in the spinal dorsal horn [25]. Thus, we determined if the effects induced by LPS are mediated by PKC activation. We found that bath perfusion of a PKC activator (PMA, 2 μM) significantly reduced amplitudes and areas of GABA currents (Figure 3A). The effects induced by PMA under such condition may result from direct activation of PKC in the recorded neuron or from indirect effects induced by activation of PKC in other cell types. To specifically address this issue, PKC in the recorded neuron was blocked by microdialyzing the PKC inhibitor (PKCI 19–30, 5 μM) [35], included in the recording glass pipette, into the recorded neuron. Recordings were made 15 minutes after rupturing the cell. Under such condition, GABAergic currents induced by the puffed GABA (100 μM) remained unchanged when LPS (1 μg/ml) was bath perfused (Figure 3B). Together with the data that the LPS-induced inhibitory effects on GABAergic receptor activities were prevented by minocycline (Figure 1B), these data indicate that LPS causes release of IL-1β from microglia, and IL-1β in turn suppresses neuronal GABA receptor activities through activating PKC in neurons.

### Presynaptic mechanisms contributed to the LPS-induced suppression of GABAergic synaptic activities

**Figure 4:**
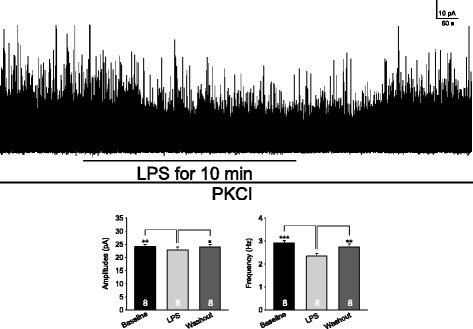
**Presynaptic mechanisms contribute to the lipopolysaccharide (LPS)-induced suppression of GABAergic synaptic activities.** Shown is the GABAergic miniature inhibitory postsynaptic currents (mIPSCs) recorded with a recording pipette filled with the intracellular solution containing the protein kinase C (PKC) inhibitor (PKCI 19–30, 5 μM) at baseline, during and after washout of LPS (1 μg/ml). Bar graphs show mean (+SE) amplitudes and frequency before, during and after washout of LPS. Number of neurons included in each group for the analysis is shown in each bar. *P < 0.05; **P < 0.01; ***P < 0.001.

To determine whether presynaptic mechanisms contribute to the LPS-induced suppression of GABAergic synaptic activities, the effect induced by LPS on postsynaptic GABA receptors was blocked by microdialyzing the recorded neuron with the PKC inhibitor PKCI (PKCI 19–30, 5 μM) included in the pipette solution. Under such condition, we first recorded GABAergic mIPSCs before and after perfusion of LPS (1 μg/ml). LPS significantly reduced the mean GABAergic mIPSC amplitudes by 5.57 ± 1.64% (n = 8, *P* < 0.01) and frequencies by 20.12 ± 1.87% (n = 8, *P* < 0.001) (Figure 4). These data suggest that presynaptic mechanisms contributed to the LPS-induced suppression of GABAergic synaptic activities.

**Figure 5:**
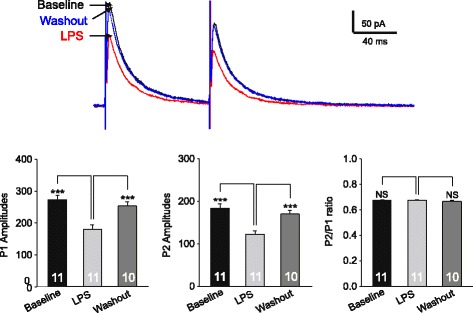
**The lipopolysaccharide (LPS)-induced suppression of gamma-amino butyric acid (GABA) synaptic activities is not ascribed to the reduced release probability of GABA from presynaptic terminals.** The raw data show two GABAergic inhibitory postsynaptic currents (IPSCs) evoked by paired pulse electrical stimuli (100 ms apart) before, during and after washout of LPS (1 μg/ml). The recordings were made with a recording pipette filled with the intracellular solution containing the protein kinase C (PKC) inhibitor (PKCI 19-–30, 5 μM). Bar graphs show mean (+SE) amplitudes of P1, P2 and P2/P1 ratios at baseline, during and after washout of LPS (1 μg/ml). ***P < 0.001; NS: no statistical significance.

The suppression of mIPSC frequencies could result from a reduction in the presynaptic GABA release probability, whereas suppression of both frequencies and amplitudes of mIPSCs more likely results from a reduction of GABA synthesis at the presynaptic site [20,36,37]. To determine if the GABA release probability at the presynaptic site is altered by LPS, we recorded two evoked GABAergic IPSCs (eIPSCs) elicited by paired pulses separated by 100 ms and analyzed the paired pulse ratio (that is P2:P1 ratio) [18,20]. P1 was the amplitude of the first evoked current and P2 was the second response amplitude measured after subtraction of the remaining P1 “tail current” [20]. An increase or decrease in the P2:P1 ratio is conventionally used to detect a decrease or increase in neurotransmitter release probability at the presynaptic site [21,38-41], including GABAergic synapses in the spinal dorsal horn [20,42]. As shown in Figure 5, although both P2 and P1 values of the GABAergic eIPSCs were reduced after bath perfusion of LPS, the P2:P1 ratios remained unchanged (0.67 ± 0.014 at baseline and 0.66 ± 0.025 during LPS perfusion, n = 11, *P* = 0.76). These results suggested that the LPS-induced suppression of GABA synaptic activities is not ascribed to the reduced release probability of GABA from presynaptic terminals. Instead, these data suggest that LPS may reduce GABAergic activities through impairing GABA synthesis.

### Reduction of GABA synthesis through the glutamate-glutamine cycle contributes to the LPS-induced suppression of GABAergic IPSCs

**Figure 6:**
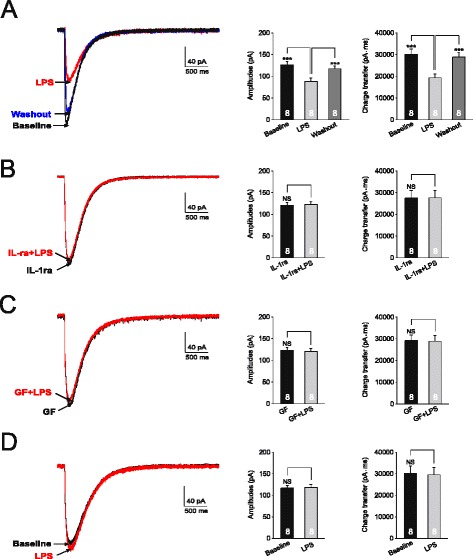
**Lipopolysaccharide (LPS) suppresses glial glutamate transporter activities through releasing IL-1β and activating protein kinase C (PKC).** Glutamate transporter currents (GTCs) were recorded from glial fibrillary acidic protein-GFP (GFAP-GFP) mice. Samples of GTCs recorded before (baseline), during and after washout of LPS (1 μg/ml) are shown in **(A)**. Samples of GTCs recorded before (baseline), during perfusion of LPS (1 mg/ml) in the presence of IL-1β receptor blocker (IL-1ra, 100 ng/ml) **(B)** or in the presence of the PKC inhbitor, GF109203X (GF, 4 μM) **(C)** are shown. Samples of GTCs recorded before (baseline), during perfusion of LPS (1 mg/ml) with a recording pipette filled with the intracellular solution containing the PKC inhibitor (PKCI 19–30, 5 μM) are shown in **(D)**. Bar graphs show the mean (+SE) amplitude and charge transfer of GTCs before (baseline), during and after washout of tested agents. ***P < 0.001; NS: no statistical significance.

We recently demonstrated that IL-1β reduces glial glutamate transporter activities [25] and the impairment of glial glutamate transporters results in a reduction of GABA synthesis through the glutamate-glutamine cycle [20]. Thus, we extrapolated that the endogenous IL-1β release induced by LPS reduces GABA synthesis by suppressing glial glutamate transporter activities. We then investigated the effects of LPS on glial glutamate transporter activities in astrocytes. The uptake of glutamate by glial glutamate transporters is accompanied by the cotransport of two to three Na^+^ with one H^+^ and the countertransport of one K^+^ [43-45]. Because of the translocation of a net positive charge during each transport cycle, glutamate uptake generates a current called glutamate transporter current (GTC) [43-46]. The size of GTC reflects the amount of transported glutamate, which has been widely used as an effective tool to study the function of glial glutamate transporters [47-49]. GTCs were recorded from GFAP-GFP transgenic mice as we described previously [25]. Astrocytes in these mice were easily identified by the expression of GFP under a fluorescent microscope. We found that LPS (1 μg/ml) bath perfusion reduced amplitudes and charge transfers of GTCs (Figure 6A). This effect was blocked in the presence of the selective IL-1β inhibitor IL-1ra (100 ng/ml) (Figure 6B). We recently showed that IL-1β reduces glial glutamate transporter activities through activating PKC. Here, we further confirmed these conclusions by determining the effect of LPS on GTCs in the presence of the PKC inhibitor GF109203X (4 μM). As expected, LPS no longer attenuated GTC amplitudes and charge transfers under such condition (Figure 6C). Furthermore, when PKC in the recorded astrocyte was blocked by microdialyzing the recorded neuron with the PKC inhibitor PKCI (PKCI 19–30, 5 μM) included in the pipette solution, GTC amplitudes (n = 8, *P* = 0.44) and charge transfers (n = 8, *P* = 0.91) were not significantly altered by LPS (1 μg/ml) bath perfusion (Figure 6D). Taking together, these data indicate that LPS suppresses glial glutamate transporter activities through releasing IL-1β and activating PKC.

**Figure 7:**
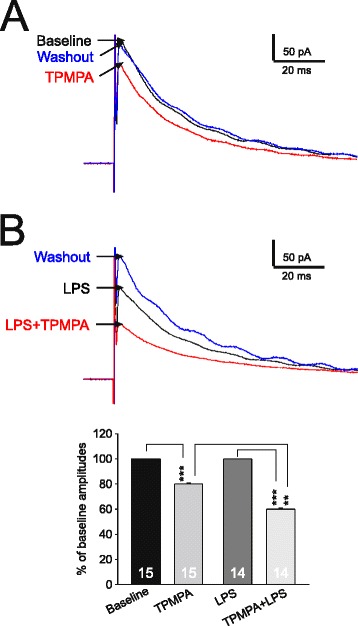
**Lipopolysaccharide (LPS) reduces the amount of gamma-amino butyric acid (GABA) release from presynaptic neurons. (A)** shows electrically evoked GABAergic evoked inhibitory postsynaptic currents (eIPSCs) at baseline, in the presence of tetrahydropyridin-4-yl methylphosphinic acid (TPMPA) (60 μM), and after TPMPA washout. **(B)** shows eIPSCs in the presence of LPS (1 μg/ml), in the presence of LPS (1 μg/ml) plus TPMPA (60 μM) and washout of both TPMPA and LPS. Note data in **(A)** and **(B)** were recorded from the same neuron. The eIPSCs were recorded with a recording pipette filled with the intracellular solution containing the PKC inhibitor (PKCI 19–30, 5 μM). The bar graph show mean (+SE) percentages of eIPSC amplitudes during perfusion of TPMPA in the absence of LPS (second bar to the left), and in the presence of LPS (the far right bar). Amplitudes prior to TPMPA perfusion in the absence (the far left bar) or presence of LPS (the second bar to the right) were used as the baseline for calculating the percentages. **P < 0.01; ***P < 0.001.

**Figure 8:**
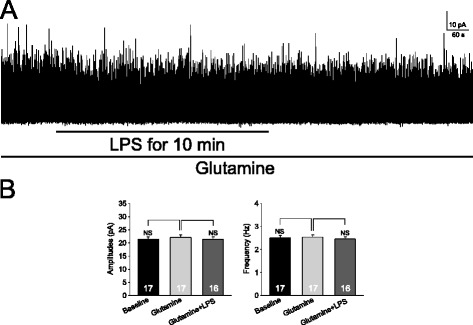
**Exogenous glutamine prevents the reduction induced by lipopolysaccharide (LPS) in GABAergic miniature inhibitory postsynaptic currents (mIPSCs). (A)** shows GABAergic mIPSCs in the presence of glutamine (1 mM): amplitudes and frequencies of mIPSCs were not changed by bath-application of LPS (1 μg/ml). The recordings were made with a recording pipette filled with the intracellular solution containing the protein kinase C (PKC) inhibitor (PKCI 19–30, 5 μM). **(B)** Bar graphs show that mean (+SE) amplitudes and frequencies of mIPSCs in the presence of glutamine (1 mM) were not significantly altered by LPS. NS: no statistical significance.

To further dissect the presynaptic mechanism underlying the changes in GABAergic synaptic activities induced by LPS, the results shown in Figures 7 and 8 were collected under the condition when the PKC inhibitor PKCI (PKCI 19–30, 5 μM) was included in the pipette solution. If the LPS-induced suppression of glial glutamate transporter activity leads to a reduction of GABA synthesis, the amount of GABA released from presynaptic terminal vesicles should be reduced. In other words, the concentration in the GABAergic synaptic cleft is reduced [18,20]. To prove this, we examined the inhibition induced by TPMPA on GABAergic IPSCs in the absence and presence of LPS. Because TPMPA is a low-affinity competitive antagonist of GABA_A_ receptors, the binding of TPMPA to GABA_A_ receptors is readily replaced by synaptic GABA neurotransmitter. When GABA_A_ receptors are exposed to TPMPA, the degree of inhibition caused by TPMPA on GABAergic IPSCs is inversely related to the concentration of GABA transients (the amount of GABA release) in the synaptic cleft [18,50]. As shown in Figure 7, the percentage of inhibition by TPMPA (60 μM) on GABAergic IPSCs in the presence of LPS (37.04 ± 1.72%, n = 14) was significantly higher (*P* < 0.01) than that in the absence of LPS (19.90 ± 0.62%, n = 15). These data indicate that inhibition induced by LPS on GABAergic IPSCs was indeed due to a decrease, at the synaptic cleft, in the amount of GABA release from presynaptic neurons.

Based on the glutamate-glutamine cycle theory (see introduction [16]), we reasoned that if the inhibitory effects induced by LPS on GABAergic IPSCs is due to its inhibition on glial glutamate transporters and subsequent inhibition of the glutamate-glutamine cycle, glutamine concentrations in the extracellular space in the presence of LPS should be reduced. These would result in a deficient glutamine supply to GABAergic neurons and a reduction in GABA synthesis. If this holds, supplementing exogenous glutamine in the recording bath should prevent the reduction induced by LPS in GABAergic eIPSCs [18,20]. As expected, in the presence of 1 mM glutamine, LPS no longer significantly altered amplitudes and frequencies of GABAergic mIPSCs (Figure 8).

**Figure 9:**
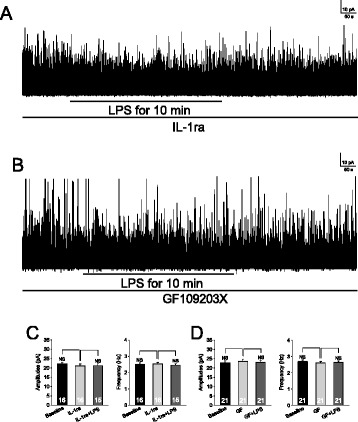
**Inhibition of protein kinase C (PKC) or IL-1β receptors blocks the inhibitory effects induced by lipopolysaccharide (LPS) on GABAergic synaptic activities at the presynaptic site.** A sample of GABAergic miniature inhibitory postsynaptic currents (mIPSCs) recorded in the presence of the IL-1β receptor blocker (IL-1ra, 100 ng/ml) **(A)** or PKC inhibitor (GF109203X, GF) **(B)** before, during, and after washout of LPS (1 μg/ml). Bar graphs show that mean (+SE) frequencies and amplitudes of GABAergic mIPSCs before (baseline), during perfusion of IL-1ra or GF109203X and further addition of LPS in the presence of IL-1ra **(C)** or GF109203X **(D)**. NS: no statistical significance.

As IL-1β and PKC mediate the suppressive effects of LPS on GTCs, we reasoned that inhibition of either IL-1β or PKC should prevent the suppressive effects of LPS on GABAergic IPSCs. As expected, we found that in the presence of the IL-1β inhibitor IL-1ra (100 ng/ml) (Figure 9A and C) or PKC inhibitor GF109203X (4 μM) (Figure 9B and D), perfusion of LPS did not alter amplitudes and frequencies of GABAergic mIPSCs. Taking together data in Figures 5, 6, 7 and 8, we conclude that: a. activation of TLR4 by LPS reduces GABAergic synaptic activities through suppressing the glutamate-glutamine cycle-dependent GABA synthesis; and b. the suppressed GABA synthesis is mediated by IL-1β, which attenuates glutamate transporter activities by activating PKC.

## Discussion

In this study, the impact of TLR4 activation by LPS on GABAergic synaptic activities in the superficial spinal dorsal horn was revealed for the first time. We demonstrate that activation of TLR4 reduces GABAergic synaptic activities through both the presynaptic and postsynaptic mechanisms. Our study provides evidence for an important modulatory role for TLR4 in GABAergic synaptic activities and demonstrates that blocking the IL-1β/PKC signaling is a powerful approach to abrogate disinhibition induced by TLR4 activation in the spinal dorsal horn.

### Roles of TLR4 in spinal nociceptive sensory processing

Emerging studies have shown glial-neuronal interactions play a critical role in the genesis of pathological pain and alteration of glial functions is a culprit causing aberrant neuronal activities in the pain signaling pathway [51-53]. Glial cells can regulate neuronal activities indirectly by releasing pro-inflammatory mediators (including pro-inflammatory cytokines, like IL-1β). It has been well known that production and release of pro-inflammatory cytokines from glial cells are controlled by TLR4. Activation of TLR4 on microglia leads to activation of NF-κB and activating protein-1 (AP-1), resulting in increased production of pro-inflammation cytokines like TNFα, IL-1β and IL-6 through transcriptional regulation [51]. Activation of TLR4 on microglia can also rapidly induce release of mature IL-1β from microglia through post-translational regulation, which involves phosphorylation of p38, ATP release and subsequent activation of P2X7 receptors [27]. While the nature of the endogenous ligands for TLR4 remains unclear, the critical role of TLR4 in the genesis of pathological pain has been demonstrated by many laboratories. Spinal TLR4 activities are increased in rats with neuropathic pain as evident by an increased expression of TLR4 accessory protein CD14 in the spinal dorsal horn of rats with nerve injury [54]. TLR4 knockout significantly attenuates behavioral hypersensitivity with decreased expression of spinal microglial markers and pro-inflammatory cytokines [4] in neuropathic rats. Pharmacological blockade of spinal TLR4 prevents [3] and rapidly reverses allodynia in rodents with neuropathic pain [55], bone cancer pain [6], or inflammatory pain induced by complete Freund’s adjuvant [7]. Activation of spinal TLR4 also plays a critical role in the transition from acute to chronic post-inflammatory mechanical hypersensitivity in arthritis [56]. Further, intrathecal or peritoneal injection of the TLR4 agonist LPS induces allodynia in mice [7] and rats [57,58]. Currently, impacts of TLR4 activation on synaptic activities in the spinal dorsal horn are unknown. Our findings provide the first evidence in the spinal cord about the synaptic plasticity induced by activation of TLR4. We show that activation of TLR4 rapidly reduces GABAergic synaptic activities in the spinal dorsal horn, and these effects were abolished when microglia activation was suppressed by the microglial suppressor minocycline or when IL-1β receptors were blocked by IL-1ra. Our findings suggest that activation of TLR4 on microglia causes release of IL-1β, which in turn suppresses GABAergic activities.

Impacts of pro-inflammatory cytokines on synaptic activities in the spinal dorsal horn have been reported. For example, release of glutamate from primary afferents is increased by exogenous TNFα [34] and IL-1β [34,59]. In spinal slices of rats with neuropathic pain, we further demonstrated that endogenous IL-1β enhances postsynaptic AMPA receptor activities through the myeloid differentiation primary response 88 (MyD88) signaling pathway, and glutamate release from the primary afferents through coupling with presynaptic NMDA receptors [59]. In addition, exogenous IL-1β reduces spontaneous GABAergic IPSCs and GABA receptor currents evoked by exogenous GABA [34]. However, the effects of endogenous IL-1β on GABAergic activities and mechanisms underlying the IL-1β-induced suppression of GABAergic activities remain unclear. Our current study demonstrated that endogenous IL-1β released from microglia following activation of TLR4 produces similar effects on GABAergic activities as those produced by exogenous IL-1β. More importantly, our study for the first time revealed the underlying mechanisms used by endogenous IL-1β released from microglia in response to activation of TLR4. At the postsynaptic site, IL-1β reduces GABA receptor functions through activating PKC in the neuron. At the presynaptic site, the content in the presynaptic GABA vesicles is attenuated by the endogenous IL-1β. This is due to a reduction of GABA synthesis through the glutamate-glutamine cycle. IL-1β reduces glutamine supply in the glutamate-glutamine cycle through suppressing glial glutamate transporters.

### Roles of glial glutamate transporters in spinal nociceptive sensory process

Another key mechanism by which glial cells control neuronal activities is through glial glutamate transporters located in astrocytes. Glial glutamate transporters regulate activation of both glutamate receptors and GABA receptors in the spinal dorsal horn. Since glutamate is not metabolized extracellularly, the clearance of glutamate released from presynaptic terminals and maintenance of glutamate homeostasis depend on glutamate transporters [52,60,61]. Glutamate transporters up-take glutamate into the cell. Glial glutamate transporters account for more than 90% of all CNS synaptic glutamate uptake [62]. Downregulation of astrocytic glutamate transporter protein expression in the spinal dorsal horn is associated with neuropathic pain induced by repeated treatments of taxol [63] or nerve injury [64-66]. Pharmacological inhibition of spinal glial glutamate transporters induces mechanical and thermal allodynia [67,68]. At the synaptic level, we demonstrated that deficient glial glutamate uptake enhances activation of AMPA and NMDA receptors, and causes glutamate to spill to the extrasynaptic space and activate extrasynaptic NMDA receptors in spinal sensory neurons [21,24,64]. Furthermore, we also show that pharmacological inhibition of glial glutamate transporters in the spinal dorsal horn results in a decrease in GABAergic synaptic activities due to impairment in GABA synthesis through the glutamate-glutamine cycle [20]. Our present study extends these findings by further demonstrating that the glutamate-glutamine cycle-dependent GABA synthesis can be reduced by endogenous IL-1β and activation of TLR4.

Despite the important role of glial glutamate transporters in spinal nociceptive processing, only a handful of studies have investigated mechanisms leading to dysfunction of spinal glial glutamate transporters function in the spinal dorsal horn. Glutamate transporter activities are decreased by increased arachidonic acid in nerve-injury-induced neuropathic rats [69]. In taxol-induced neuropathic rats, nitration of glial glutamate transporters by peroxynitrite reduces glial glutamate transporter function [63]. Suppression of glial activation with minocycline [65] or propentofylline [70] in rats with nerve injury ameliorates protein expression of glial glutamate transporters in the spinal dorsal horn. Recently, we demonstrated that pharmacological inhibition of glycogen synthase kinase 3β prevents and reverses the attenuated protein expression of glial glutamate transporters in the spinal dorsal horn and allodynia in rats receiving paclitaxel [71] and rats with partial sciatic nerve ligation [72]. These are concurrently associated with suppression of astrocytic activation and production of IL-1β [71,72]. IL-1β reduces glial glutamate transporter activities through promoting endocytosis of both GLT-1 and GLAST [25]. Given that activation of TLR4 is critical to the genesis of many pathological pain conditions, it is conceivable that suppression induced by TLR4 activation of glial glutamate transporter activities and GABAergic synaptic activities contributes to abnormal neuronal activation under these conditions.

### Regulation of GABAergic activities in the spinal dorsal horn

The superficial spinal dorsal horn is a first station for processing nociceptive inputs from peripheral nociceptive Aδ and C fibers. Evidence has been reported that weakening of GABAergic synaptic strength in this area contributes to the genesis of neuropathic pain. Extracellular GABA levels in the lumbar dorsal horn have been shown to be decreased by nerve injury [73]. Amplitudes and frequencies of GABA_A_ receptor-mediated IPSCs in neurons in the superficial dorsal horn of neuropathic rats are reduced [15]. Spinal application of GABA agonists attenuates mechanical allodynia and thermal hyperalgesia induced by nerve injury [74]. Strength of GABAergic synaptic activities at the spinal dorsal horn is regulated at both the presynaptic and postsynaptic sites. Through modulating the release probability of GABA neurotransmitters, activation of presynaptic A1 adenosine-receptor adenosine or GABA_B_ receptors at the presynaptic terminals suppresses GABA release [22,75], whereas activation of neuronal acetylcholine receptors [76] or activation of M3 muscarinic acetylcholine receptors [77] increases GABA release from the presynaptic terminals. Our present study revealed that the reduction of GABA synthesis at the presynaptic site induced by endogenous IL-1β is an important mechanism leading to disinhibition under the condition when TLR4 is activated. At the postsynaptic site, inhibitory currents induced by activation of GABA receptors are reduced by the downregulation of the K^+^-Cl^−^cotransporter KCC2, which disrupts Cl^−^ homeostasis in neurons [14]. Our findings that postsynaptic GABA receptor activities are suppressed by LPS through activation of PKC in the neuron provide a novel mechanism controlling GABAergic synaptic strength in the spinal dorsal horn under neuroinflammation.

## Conclusions

Our present study has demonstrated that activation of TLR4 in the spinal dorsal horn with LPS results in release of IL-1β from microglia. IL-1β in turn suppresses GABAergic synaptic activities through both presynaptic and postsynaptic mechanism. Attenuation of glial glutamate transporter activities and activation of PKC are implicated in the LPS-induced suppression of GABAergic synaptic activities. These findings shed light on understanding synaptic plasticity induced by activation of TLR4 under neuroinflammation and identify GABA receptors, glial glutamate transporters, IL-1β and PKC as therapeutic targets to abrogate abnormal neuronal activities following activation of TLR4 activation in the spinal dorsal horn.

## Abbreviations

aCSF: artificial cerebrospinal fluid
ANOVA: analysis of variance
CNS: central nervous system
EDTA: ethylenediaminetetraacetic acid
eIPSCs: evoked GABAergic IPSCs
GABA: Gamma-amino butyric acid
GFAP: glial fibrillary acidic protein
GFP: green fluorescent protein
GLAST: glutamate–aspartate transporter
GLT-1: glial glutamate tansporter-1
GTC: glutamate transporter current
IL-1β: interleukin-1β
IL6: interleukin-6
IPSCs: inhibitory postsynaptic currents
LPS: lipopolysaccharide
mIPSCs: miniature inhibitory postsynaptic currents
MyD88: the myeloid differentiation primary response 88
PKC: protein kinase C
PKCI: PKC inhibitor
PMA: phorbol 12-myristate 13-acetate
SE: standard error
TPMPA: tetrahydropyridin-4-yl methylphosphinic acid
TLR4: toll like receptor 4
TNFα: tumor necrosis factor α
